# The mRNA N^6^-Methyladenosine Response to Dehydration in *Xenopus laevis*

**DOI:** 10.3390/ani14223288

**Published:** 2024-11-15

**Authors:** Saif Rehman, Mackenzie Parent, Kenneth B. Storey

**Affiliations:** Department of Biology, Carleton University, Ottawa, ON K1S 5B6, Canada

**Keywords:** epigenetics, dehydration, N^6^-methyladenosine, mRNA, metabolic rate depression, *Xenopus laevis*

## Abstract

The African clawed frog, *Xenopus laevis*, exhibits remarkable adaptations to survive in its arid habitat, including behavioral and metabolic changes during periods of drought. During extreme dehydration, *X. laevis* undergoes estivation, a state characterized by increased urea and ammonia levels, depression of the metabolic rate, and tissue hypoxia. To understand the molecular mechanisms underlying these adaptations, we investigated the potential role of N6-methyladenosine (m^6^A), a widespread mRNA modification, in *X. laevis* during extreme dehydration. We analyzed the protein levels of key components in the m^6^A pathway, including writers (METTL3, METTL14, and WTAP), erasers (ALKBH5 and FTO), and readers (SRSF3, YTHDF1, YTHDF2, YTHDF3, and eIF3a), in the liver and kidneys of control frogs and frogs that had lost 35 ± 0.93% of their total body water. The relative protein levels generally decreased or remained unchanged, with the exception of YTHDF3, which depicted a protein level increase in the liver. Notable changes included eIF3a, which was downregulated by 26 ± 8% and 80 ± 8% in the dehydrated liver and kidney tissues, respectively. Additionally, the total m^6^A increased by 353 ± 30% and 177 ± 17% in dehydrated liver and kidney RNA samples, respectively. This study highlights the importance of epigenetic mechanisms in stress tolerance and provides a foundation for further exploration of the role of epigenetics in dehydration tolerance.

## Simple Summary

Adapting to severe dehydration poses significant challenges for wildlife, particularly for the African clawed frog, *Xenopus laevis*, which relies on estivation to survive dry seasons. By examining the protein levels of key components in the m^6^A modification pathway, we found that while most m^6^A-related proteins responsible for adding, removing, or interpreting m^6^A marks decreased during dehydration, the total amount of m^6^A on mRNA increased substantially in the liver and kidneys. This increase may serve as a rapid, reversible way for *X. laevis* to adjust gene activity and conserve energy under stressful conditions. Although many proteins involved in energy-intensive processes were downregulated, one specific m^6^A reader protein showed increased levels in the liver, potentially aiding in selective gene expression during dehydration. Our findings suggest that these molecular adjustments help frogs survive prolonged water scarcity, enhancing our understanding of how animals manage extreme environmental stress.

## 1. Introduction

The African clawed frog, *Xenopus laevis*, inhabits stagnant grassland ponds of arid/semi-arid regions. With this territory comes the necessity to survive through periods of drought that are common to these environments. *X. laevis* employs behavioral, as well as metabolic adaptations, in order to survive periods of seasonal drought. To conserve their body water, frogs will burrow into the cool mud of drying lakebeds [1]. When the behavioral adaptations begin to fail, frogs enter a state known as estivation, in which they may lose up to ~35% of their body mass due to water evaporation [2,3]. Estivation is also characterized by increases in urea and ammonia levels in tissues, as well as a strong depression of the metabolic rate [4,5].

Uniquely, *X. laevis* does not exhibit extreme changes in the metabolic rate during estivation but must still make changes to its metabolism in order to survive seasonal water shortages [2,6]. As a result of dehydration, *X. laevis* tissue ammonia and urea levels increase, blood viscosity increases, and maximal heart rate decreases, which lead to tissues and vital organs becoming hypoxic [7,8]. In response to oxygen supply problems caused by dehydration, ATP production shifts toward anaerobic glycolysis [4]. This shift to anaerobic metabolism is supported by multiple studies providing data on enzyme kinetics that suggest that key glycolytic enzymes are differentially regulated to support increased anaerobic ATP production [3,9,10].

Several studies have found potential mechanisms through which these changes could be enacted, involving changes in signalling cascades and other dynamic reversible regulatory mechanisms such as DNA methylation, histone modifications, and miRNA action [11,12,13]. Organisms such as 13-lined ground squirrels (*Ictidomys tridecemlineatus*), wood frogs (*Lithobates sylvatica*), plateau pikas (*Ochotona curzoniae*), and gray mouse lemurs (*Microcebus murinus*), among others, have shown these metabolic and molecular adaptations to play potentially vital roles in environmental stress survival [1,14,15,16,17,18,19]. One method that may potentially be involved in modulation of gene expression but has rarely been explored is the modification of messenger RNA (mRNA) transcripts. There are a number of known modifications that mRNA transcripts can undergo, the most abundant and widespread being N6-methyladenosine (m^6^A) [20,21]. Limited studies of m^6^A involvement in the stress response have depicted its possible involvement in stress survival and its possible ability to rapidly respond to environmental changes [22,23]. This includes the metabolic freeze-tolerant response of *L. sylvatica*, metabolic reprogramming of senescent leaves, as well as possibly the psychiatric stress response in humans [24,25,26].

The m^6^A modification is added to mRNAs by the action of the methyltransferase complex made up of the core subunits N6-adenosine methyltransferase like 3 (METTL3) and N6-adenosine methyl transferase like 14 (METTL14), as well the regulatory subunit Wilms’ tumor-associated protein (WTAP) [27]. The reaction occurs with the donation of a methyl group from the universal methyl donor S-adenosyl methionine (SAM). The METTL3 subunit is the catalytically active subunit, whereas METTL14 has very low catalytic activity by itself and WTAP lacks a catalytically active domain [28,29]. The reverse of the reaction described above, the demethylation reaction, is mediated by two enzymes: AlkB homology 5 (ALKBH5) and the fat mass and obesity-associated protein (FTO) [30,31]. The demethylase enzymes are essential for efficiency in mRNA metabolism [31].

The m^6^A modification, via the action of binding proteins termed ‘readers’, can have a variety of effects on mRNA transcripts. The main binding proteins involved in reading the m^6^A mark are the YTH-domain-containing family proteins (YTHDF/C), that bind the mark via recognition of a conserved domain with three key tryptophan residues [32]. Binding of the protein YTHDF1 promotes translational efficiency by facilitating interactions with translation-initiation machinery such as eIF3a that promotes cap-independent translation (eukaryotic initiation factor 3a) [20]. YTHDF3 also increases translational efficiency in a cooperative manner with YTHDF1 [33]. Binding of YTHDF2 to the m^6^A mark leads to localization of mRNAs to the processing bodies where they are eventually degraded [34]. The modification can also impact splice variants and nuclear export through interactions with the proteins YTHDC1 and SRSF3/10 [35]. SRSF3 can outcompete SRSF10 in splice variant regulation and solely dictates nuclear export through an interaction with nuclear export factor 1 (NXF1) [36].

The m^6^A modification is clearly an important form of transcriptional regulation and plays an essential role in the metabolism of mRNAs, indicating that it could be an important component of reversible gene modulation. The mark is currently underexplored in terms of stress tolerance and unexplored in conjunction with dehydration. This study aims to analyze the effects of tissue dehydration on the m^6^A pathway proteins in *Xenopus laevis*. It is expected that, as is the case with many energy-expensive pathways during high stress, m^6^A pathway proteins will experience a decrease in protein levels during dehydration to maintain a metabolic rate depression (MRD) [4,37]. The progress made in this study could provide insight into the molecular mechanisms that contribute to the metabolic rearrangement that occurs in *X. laevis* during periods of high dehydration brought on by dry seasonal droughts.

## 2. Materials and Methods

### 2.1. Animal Treatments and Tissues

Male adult African clawed frogs (*X. laevis*) were purchased from the University of Toronto. Upon delivery, the frogs were placed in tanks of dechlorinated water at 22 ± 1 °C for 3 weeks before experiments began. Frogs were fed between 3 and 4 pellets of CU Adult Frog diet (PMI Nutrition International) three times per week and their water was changed after each feeding. Animals were then separated randomly into control and dehydration treatment groups and were not fed again (*n* = 5 per group). Animals in the control group were maintained under the conditions outlined above, whereas animals of the dehydration group were placed into dry containers at 22 °C and allowed to lose water by evaporation across the skin. These animals were weighed twice daily over the course of several days until the desired water loss was achieved. The percentage body water loss was calculated using the following equation:

$$\%Body\;water\;loss=\frac{m_{i}-m_{d}}{m_{i}\cdot BWC_{i}}$$

where $m_{i}$ represents the initial mass, $m_{d}$ represents dehydrated mass, and $BWC_{i}$ is the initial body water content. The initial body water content was determined to be 0.74 ± 0.002 g H_2_O per g body mass [10]. The percentage body water loss of dehydrated animals was 35 ± 0.93%, which is the limit of what is tolerable by this species [38]. Animals were euthanized by pithing and tissues were quickly dissected before being flash frozen in liquid nitrogen and stored at −80 °C until needed. All protocols were approved by the Carleton University Animal Care Committee (protocol #106936) and conformed with the guidelines of the Canadian Council on Animal Care.

### 2.2. Total Protein Extraction

Total protein was extracted from frog liver and kidney tissues from control and high-dehydration samples (*n* = 5 independent biological replicates). The samples were placed in ice-cold 1X Lysis buffer (Cell Signalling, Danvers, MA, USA; Cat # 43-040) with 1 mM Na_3_VO_4_, 10 mM NaF, 10 mM β-glycerophosphate, and a 10 µL/mL protease inhibitor cocktail (BioShop, Burlington, ON, Canada; PIC002) at a 1:5 *W*:*V* ratio. Samples were homogenized immediately after being added to the buffer using a Polytron homogenizer for 10–30 s. Samples were then incubated on ice for 30 min with vortexing every 10 min. After incubation, the samples were centrifuged at 10,000× *g* for 30 min at 4 °C. Supernatants were collected and transferred to new tubes. Protein concentration was measured using a Bio-Rad protein assay (Bio-Rad, Singapore, Singapore; Cat #500-0006) with bovine serum albumin used for a standard curve. Sample protein concentrations were then standardized by the addition of small aliquots of homogenization buffer. The samples were stored at −80 °C until needed.

### 2.3. SDS-PAGE

As previously described [39], standardized total protein extracts were mixed 1:1 *v*:*v* with 2× loading buffer (100 mM Tris-HCl, 4% *w*/*v* SDS, 0.2% *w*/*v* bromophenol blue, 10% *v*/*v* 2-mercaptoethanol, and 20% *v*/*v* glycerol). Samples were then boiled for 5 min on a water bath and allowed to cool before being stored at −80 °C until use. For electrophoresis, sample aliquots containing 15, 20, or 40 µg protein (depending on the target being assessed) from both conditions (control and dehydrated) were loaded onto 5% acrylamide upper stacking gels sitting atop resolving gels at 8, 10, 12, or 15% depending on the molecular weight of the target protein (acrylamide/bis-acrylamide ratio 29.2:0.8; *w*:*w*). The first lane was loaded with PiNK Plus Prestained Protein Ladder (Froggabio, Concord, ON, Canada: PM005-0500) or BLUeye Prestained Protein Ladder (Froggabio: PM007-0500) depending on the size of the protein of interest. Electrophoresis was performed at a constant voltage of 180 V at room temperature in Tris glycine running buffer (0.25 M Tris-base, 0.035 M SDS, and 2.45 M glycine).

Subsequently, proteins were transferred from polyacrylamide gels to polyvinylidene difluoride (PVDF) membranes (immobilon-P transfer membrane, Millipore corp. Bedford, MA, USA) at a constant current of 160 mA at 4 °C for 60 to 120 min depending on the size of the protein of interest. Transfer was performed in transfer buffer (25 mM Tris (pH 8.5), 192 mM glycine, and 20% *v*/*v* methanol). Washing steps were carried out with TBST (150 mM NaCl, 20 mM Tris pH 7.5, 0.05% Tween-20).

### 2.4. Immunoblotting

Following transfer, PVDF membranes were washed for 3 × 5 min with shaking at room temperature for all washing steps. Membranes were blocked using between 5 and 10% non-fat milk in TBST for 30 min. Membranes were washed again prior to incubation with primary antibody (1:1000 *v*:*v* TBST) on a shaking platform overnight at 4 °C (Table A1). After incubation with the primary antibody, blots were washed twice with TBST and incubated with an anti-rabbit secondary antibody (diluted 1:2000 in TBST) for 30 min at 21 °C. Following three washes of 10 min each with TBST, protein bands were visualized by adding 1.4 mL of enhanced chemiluminescence reagent (Pierce, Los Angeles, CA, USA). Chemi-Genius Bio-Imaging system and Gene Tools software (version #4.3.8.0) from Syngene, MD, USA were used to visualize the membranes. To normalize protein loading, the Coomassie blue total protein staining method was employed. Total protein on the PVDF membrane was stained for 30 min with Coomassie blue solution (0.25% *w*:*v* Coomassie Brilliant Blue R, 50% *v*:*v* methanol, 7.5% *v*:*v* acetic acid), which was also quantified using the ChemiGenius Bio-Imaging system.

### 2.5. Data Quantification

Protein bands were standardized against Coomassie-blue-stained PVDF membranes after imaging [40]. Data for each experimental condition are presented as the mean ± standard error of the mean (SEM) from five samples obtained from different animals. The fold change was determined for dehydrated samples assigning the control value as 1. Statistical analysis was conducted using a Student’s *t*-test (*p* < 0.05).

### 2.6. Demethylase Activity

Demethylation of m^6^A demethylases (ALKBH5 and FTO) was determined with the Epigenase Demethylase Activity/Inhibition Assay Kit (Epigentek, Farmingdale, NY, USA: P-9013) following the manufacturer’s instructions. The kit uses an m^6^A substrate attached to a microplate to detect un-demethylated m^6^A through specific antibodies, allowing it to quantify enzyme activity based on signal intensity. A lower signal intensity corresponds to higher enzyme activity. Briefly, the protein concentration was optimized using a pooled sample of control and high-dehydration total protein isolates. The plate was set up with 20 µg of total protein for both the control and high-dehydration samples (*n* = 4). The samples were incubated on the plate for 90 min before being washed with wash buffer. The plate was then incubated with a capture antibody, detection antibody, and enhancer antibody for 60 min, 30 min, and 30 min, respectively. After washing the plate in wash buffer 5 times, the developer solution was added, and the colour change was observed for 5 min before the reaction was stopped using the stop solution. The plate was read at 450 nm with an optional reference wavelength of 655 nm using a Multiskan spectrophotometer (Thermo Electron Corporation, Waltham, ME, USA).

### 2.7. Total RNA Extraction

Total RNA was extracted from frog liver and kidney tissues of control and high-dehydration conditions (*n* = 5 independent biological replicates). Approximately 100 mg of sample was placed in 500 µL of ice-cold Tri Reagent (Molecular Research Center: TR118) before being homogenized using a polytron homogenizer for 10–30 s. After homogenization, another 500 µL of Tri Reagent was added and the mixture was incubated for 5 min at room temperature. Then, 200 µL of chloroform was added and the samples were shaken for 15 s before being incubated for 10 min at room temperature. After incubation, the samples were centrifuged at 10,000 rpm for 15 min at 4 °C. Following centrifugation, the upper aqueous layer was collected and transferred to new tubes. A 500 µL aliquot of isopropanol was then added to each sample before mixing and incubating for 10 min at room temperature. After incubation, the samples were centrifuged at 12,000 rpm for 15 min at 4 °C. The supernatant was discarded, and the samples were washed with 1 mL of 70% ethanol prepared in DEPC-treated water, before centrifuging at 7500 rpm for 5 min. DEPC-treated water was made by adding 1:1000 DEPC (diethylpyrocarbonate) to distilled water and stirring at room temperature for 24 h. The supernatant was then discarded, and samples were allowed to air dry for 10 min. The dried pellets were resuspended in 20–50 µL of DEPC-treated water and stored at −80 °C until needed.

### 2.8. Dot Blot m^6^A Quantification

Aliquots of RNA samples were diluted to working concentrations before being placed in a boiling water bath for 3 min. Immediately after boiling, the samples were placed on ice. Aliquots containing 100–3000 ng of RNA were dotted onto a dry nylon membrane by pipette. After dotting, the membrane was left to dry for 20 min before being placed in a UV crosslinker (SGLinker, Alcobendas, Madrid, Spain: Ref.: 13-70010 XX) set at 125 mJoule/cm^2^ at 254 nM. After crosslinking, the membranes were immunoblotted as detailed in Section 2.3.

## 3. Results

Relative protein levels of key components in the m^6^A pathway were analysed from both liver and kidney samples of *X. laevis*, comparing control and dehydrated conditions. Analysis included the major enzymes in the methyltransferase (writer) complex, the demethylases (erasers), and some of the important m^6^A binding proteins (readers). The analysis of ‘writer’ included METTL3, METTL14, and WTAP. The ‘erasers’ were ALKBH5 and FTO. The ‘readers’ were SRSF3, YTHDF1, YTHDF2, and YTHDF3.

### 3.1. Writers

**Figure 1:**
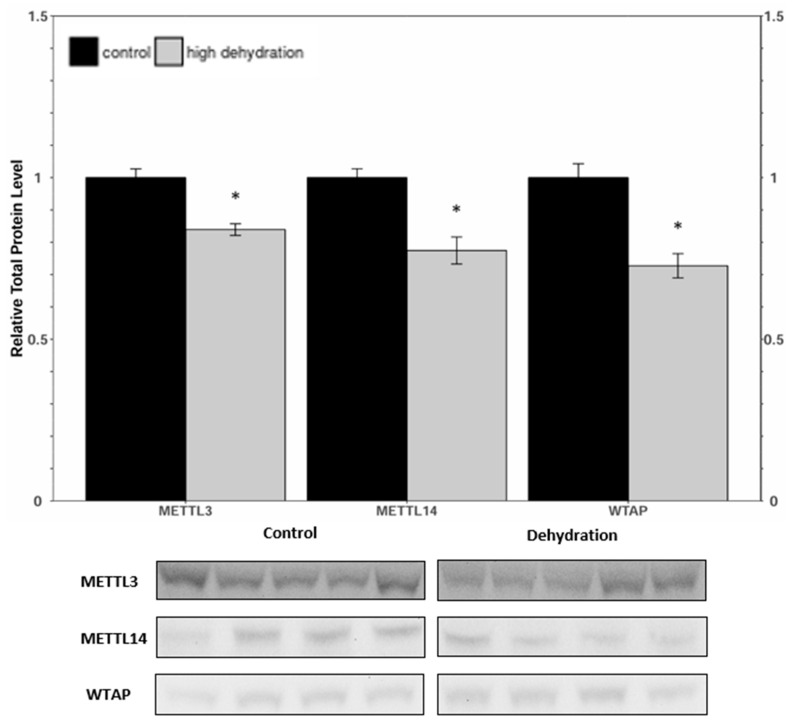
Relative protein expression levels of METTL3, METTL14, and WTAP in livers of control and dehydrated *X. laevis*, as determined by Western blotting. Corresponding bands from immunoblots are shown below the histogram. Data are mean ± SEM, *n* = 4–5 independent trials on samples from different animals. Data were analyzed using a Student’s *t*-test; asterisks indicate values that are significantly different from the corresponding control (*p* < 0.05).

**Figure 2:**
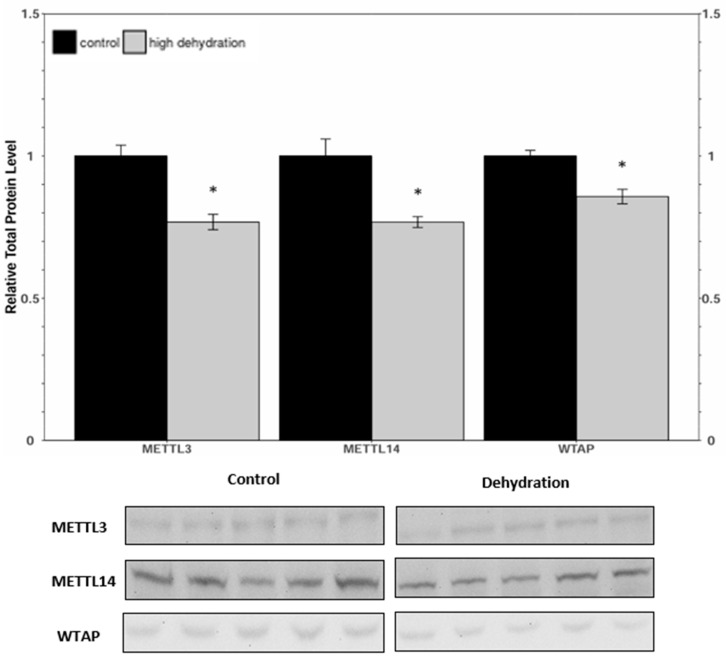
Relative protein expression levels of METTL3, METTL14, and WTAP in kidneys from control and dehydrated *X. laevis*, as determined by Western blotting. Corresponding bands from immunoblots are shown below the histogram. Data are mean ± SEM, *n* = 5 independent trials on samples from different animals. Data were analyzed using a Student’s *t*-test; asterisks indicate values that are significantly different from controls (*p* < 0.05).

Western blots were conducted to analyse the relative protein levels of the writer complex proteins (METTL3, METTL14, and WTAP) in liver and kidney tissues from control and dehydrated frogs (Figure 1 and Figure 2). In the livers of 35 ± 0.93% dehydrated frogs, levels of METTL3, METTL14, and WTAP were all found to decrease, falling by 16 ± 4%, 23 ± 6%, and 27 ± 6%, respectively, relative to the controls. In the kidneys from dehydrated frogs, METTL3, METTL14, and WTAP were decreased by 23 ± 5%, 23 ± 7%, and 14 ± 4%, respectively, relative to the controls.

### 3.2. Erasers

**Figure 3:**
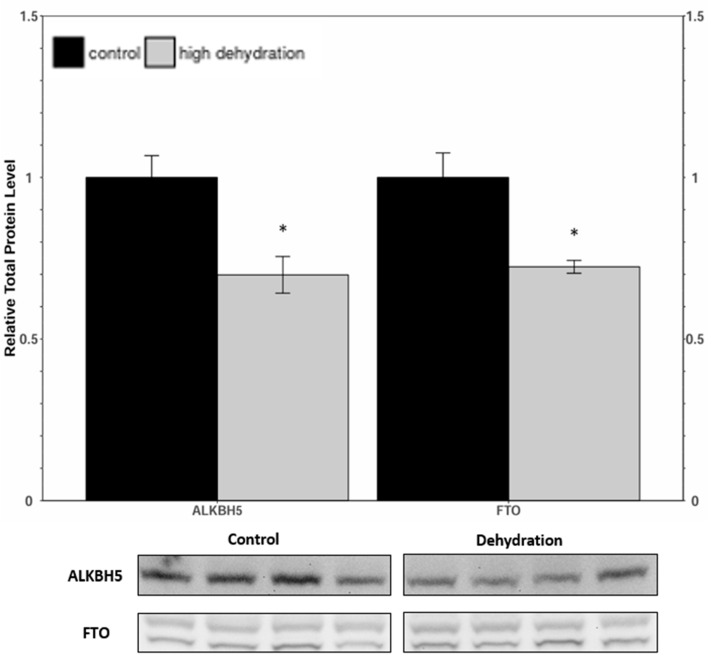
Relative protein expression levels of ALKBH5 and FTO in livers from control and dehydrated *X. laevis*, as determined by Western blotting. Corresponding bands from immunoblots are shown below the histogram. Data are mean ± SEM, *n* = 4 independent trials on samples from different animals. Data were analyzed using a Student’s *t*-test; asterisks indicate values that are statistically significantly different from controls (*p* < 0.05).

**Figure 4:**
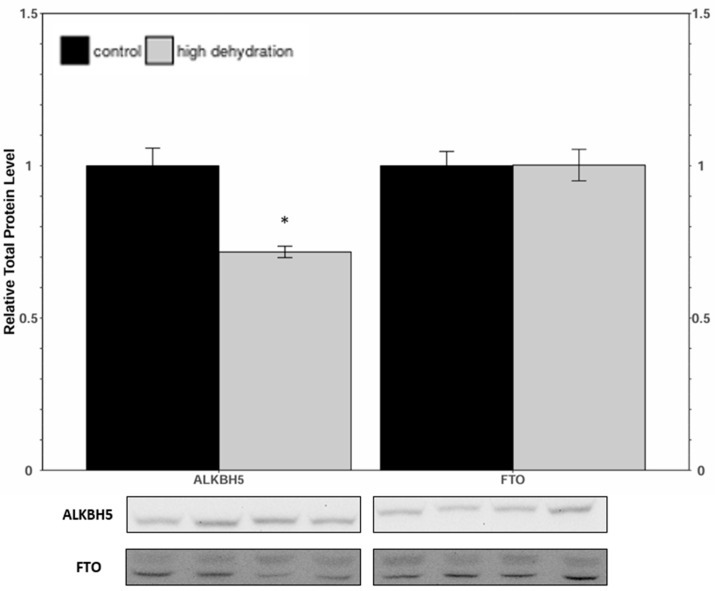
Relative protein expression levels of ALKBH5 and FTO in kidneys of control and dehydrated *X. laevis*, as determined by Western blotting. Corresponding bands from immunoblots are shown below the histogram. Data are mean ± SEM, *n* = 4 independent trials on samples from different animals. Data were analyzed a Student’s *t*-test; asterisks indicate that are statistically significantly different from controls (*p* < 0.05).

Western blots were performed to analyse the relative protein levels of the eraser proteins ALKBH5 and FTO from control and dehydrated conditions of liver (Figure 3) and kidney (Figure 4) tissues. In dehydrated livers, ALKBH5 and FTO were found to decrease by 30 ± 9% and 28 ± 8%, respectively, relative to the controls. In dehydrated kidneys, ALKBH5 was found to decrease by 28 ± 7% relative to the controls, whereas the FTO content did not change significantly.

### 3.3. Readers

**Figure 5:**
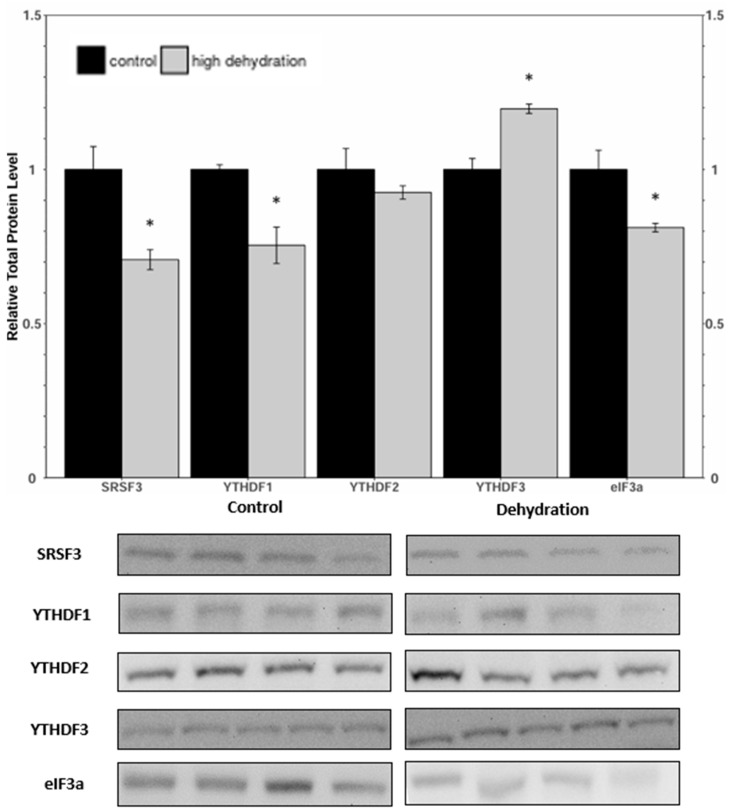
Relative protein expression levels of SRSF3, YTHDF1, YTHDF2, YTHDF3, and eIF3a in livers of control and dehydrated *X. laevis*, as determined by Western blotting. Corresponding bands from immunoblots are shown below the histogram. Data are mean ± SEM, *n* = 4–5 independent trials on samples from different animals. Data were analyzed using a Student’s *t*-test; asterisks represent values that have statistically significant changes compared with controls (*p* < 0.05).

**Figure 6:**
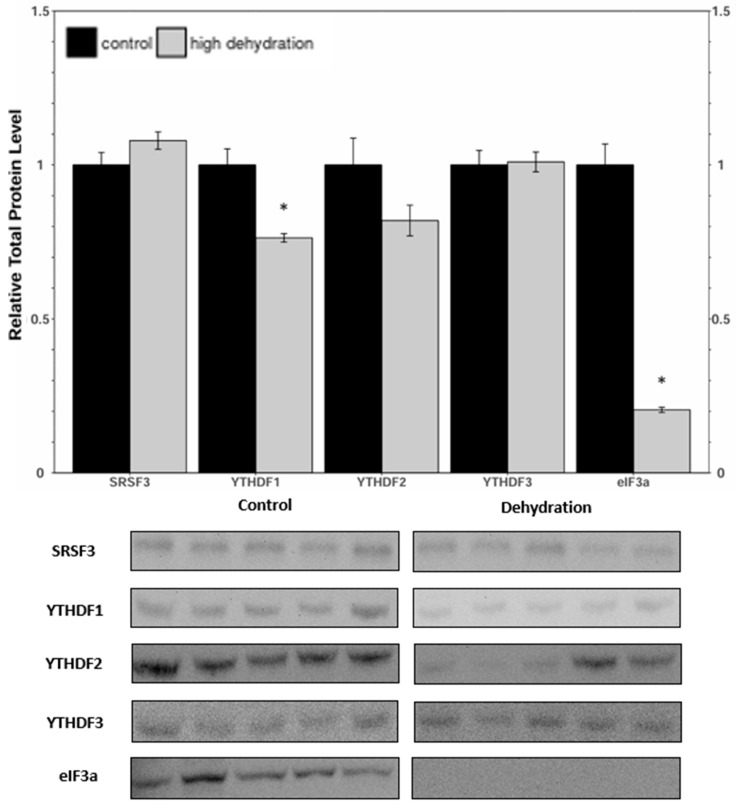
Relative protein expression levels of SRSF3, YTHDF1, YTHDF2, YTHDF3, and eIF3a in kidneys from control and dehydrated of *X. laevis*, as determined by Western blots. Corresponding bands from immunoblots are shown below the histogram. Data are mean ± SEM, *n* = 5 independent trials on samples from different animals. Data were analyzed using a Student’s *t*-test; asterisks represent values that have statistically significant changes compared with controls (*p* < 0.05).

Western blots were performed to analyse the relative protein levels of the reader proteins SRSF3, YTHDF1, YTHDF2, YTHDF3, and eIFf3a under control and dehydrated conditions of liver and kidney (Figure 5 and Figure 6) tissues. In the livers from dehydrated frogs, SRSF3, YTHDF1, and eIF3a had decreased by 29 ± 9%, 30 ± 7%, and 26 ± 8%, respectively, relative to the controls. YTHDF2 showed no statistically significant change, and YTHDF3 increased by 20 ± 4%. In the kidneys of dehydrated frogs, SRSF3 and YTHDF3 showed no statistically significant change, whereas YTHDF1 and eIF3a decreased by 24 ± 6%, and 80 ± 8%, respectively. Kidney YTHDF2 protein levels exhibited a non-significant downward trend.

### 3.4. Demethylase Activity

**Figure 7:**
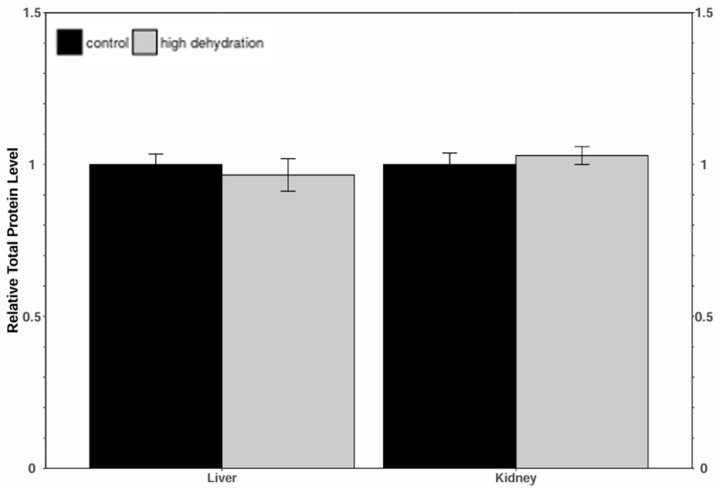
Relative m^6^A demethylase enzyme activity in liver and kidney tissues of *X. laevis* from control and high-dehydration conditions. Data are mean ± SEM, *n* = 4 independent trials on samples from different animals. Independent trials were taken as the mean of duplicate measurements. Data were analyzed using a Student’s *t*-test.

The relative m^6^A demethylase enzyme activity was determined in control vs. high-dehydration *X. laevis* liver and kidney tissues. No statistically significant difference in activity was measured between the conditions in either tissue (Figure 7).

### 3.5. Total m^6^A Quantification

**Figure 8:**
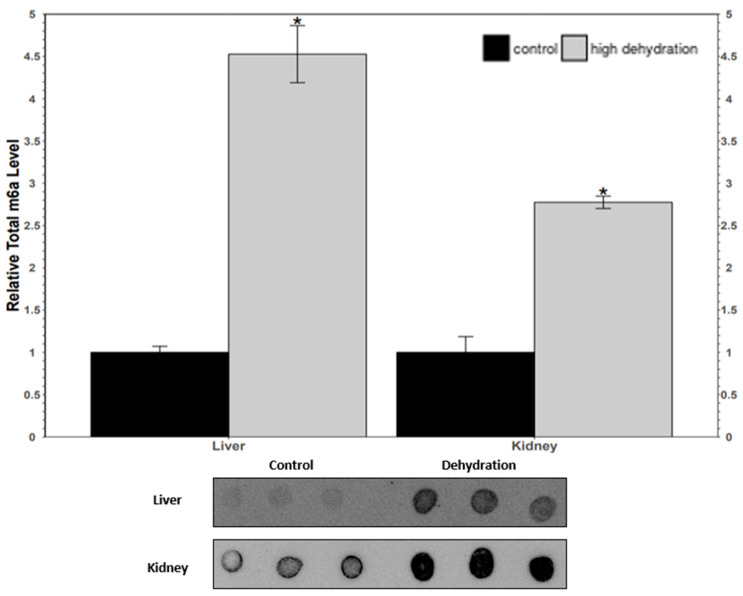
Relative total m^6^A content in total RNA samples from *X. laevis* liver and kidney tissues comparing control vs. high-dehydration conditions. Data are mean ± SEM, *n* = 3 independent trials on samples from different animals. Data were analyzed using a Student’s *t*-test; asterisks represent values that have statistically significant changes compared with controls (*p* < 0.05).

The relative m^6^A content was assessed in control vs. high-dehydration *X. laevis* liver and kidney total RNA samples. The total m^6^A was found to increase by 353 ± 30% in liver RNA samples, and by 177 ± 17% in kidney RNA samples (Figure 8).

## 4. Discussion

The loss of large amounts of body water poses many challenges to biological systems that require water for all of their vital processes. Strategies, such as estivation, have been developed over evolutionary time to help animals adapt to such conditions [8]. Entering a state of estivation requires an organism to make significant changes to their metabolism, which must continue to meet their energetic needs during dormancy while also being rapidly reversible. Modification of mRNA transcripts, specifically m^6^A, is a potential method for such regulation because of its ability to transiently modify gene expression in an easily reversible manner. This modification is also involved in all facets of mRNA metabolism, having an ability to modulate the translation, stability, splicing, and localization of transcripts [41]. A native of sub-Saharan Africa where the year is divided into wet and dry seasons, *X. laevis* can survive extreme dehydration (as much as 35% of total body water lost) for extended periods of time by burrowing into wet mud as it dries, without experiencing permanent tissue damage. Adaptations such as a greater reliance on ATP production by anaerobic pathways, accumulation of urea/ammonia, and modulation of blood flow to vital tissues aid survival [2,6,9,38]. To elucidate the potential involvement of m^6^A in these adaptations, the levels of m^6^A pathway proteins were measured in control vs. highly dehydrated *X. laevis* liver and kidney tissues. Additionally, the activity of m^6^A erasers (FTO, ALKBH5) and the total m^6^A content were also measured in the same tissue.

Levels of METTL3, METTL14, and WTAP were all significantly downregulated in response to dehydration in both liver and kidney (Figure 1 and Figure 2) tissues. However, despite these lower levels of protein content, total relative m^6^A levels increased strongly by ~4.5-fold in liver and ~2.6-fold in kidney tissues (Figure 8). This increase in total m^6^A could indicate that the activity of the writer complex is being positively modulated in some way other than protein expression. Modulation of the activity of these enzymes could be due to reversible allosteric modifications such as phosphorylation/dephosphorylation or interaction with small-molecule ligands (e.g., ATP, GTP, cAMP). Interestingly, the levels of FTO and ALKBH5 were reduced in livers from dehydrated frogs, whereas only ALKBH5 showed a significant downregulation in the kidneys of dehydrated frogs (Figure 3 and Figure 4). No change in demethylase activity was observed in either liver or kidney tissues (Figure 7). Studies have shown that demethylation of m^6^A is catalyzed preferentially by FTO, and that FTO shows high specificity for m^6^A over other RNA methyl group modifications [30,42]. The downregulation of m^6^A demethylases could also provide an explanation for the drastic upregulation of total m^6^A, in the absence of an increase in writer complex proteins.

There was a large significant downregulation under dehydrating conditions of the translation-initiation enzyme, eIF3a, in both liver and kidney tissues. The eIF3a subunit is an important component of translation initiation and the downregulation of this protein supports the total trend of downregulation in protein content over the pathway [43,44]. Eif3a is also an important factor for the normal progression of the cell cycle, most likely due to its involvement in protein translation regulation in the cell cycle [45]. Metabolic rate depression is characterized by the downregulation of nonessential genes and processes, especially those that are very energetically costly. It stands to reason that, given that the cell cycle is a costly ATP-dependant process, it would be suppressed and/or arrested in response to extreme dehydration. The downregulation of eIF3a could be an important part of the global downregulation of protein synthesis for the conservation of energy and water during the extreme dehydration of *X. laevis*. Downregulation of eIF3a was much greater in the kidneys than in the liver. This supports evidence found in other studies that demonstrates the role of the liver in maintaining energy levels during extreme dehydration [7]. YTHDF1 was also downregulated in liver and kidney tissues (Figure 5 and Figure 6), suggesting downregulation of reader-mediated mRNA translation. It has been observed that *X. laevis* undergoes a shift in ATP production from aerobic to anaerobic metabolism during extreme dehydration [3,46]. It has also been reported that the liver shifts away from glycogenolysis in favour of glucose export during dehydration in the wood frog, *Rana sylvatica* [47].

YTHDF2 did not change significantly in the kidney or liver samples from dehydrated frogs (Figure 5 and Figure 6). Interestingly, there was a significant increase in YTHDF3 levels in the liver tissue (Figure 5). Though an exact function of YTHDF3 has not yet been elucidated, it has been reported to work synergistically with either YTHDF1 or YTHDF2 to facilitate translation or accelerate decay [48]. Maintenance of YTHDF2 levels in liver and kidney tissues could be involved in targeted degradation of transcripts that are downregulated in response to dehydration, such as those associated with aerobic metabolism and cell cycle progression [24,49]. The upregulation of YTHDF3 then may work synergistically with YTHDF2 to accelerate the decay of key mRNA targets [49]. Analysis of the degradation pathways (CCR4/NOT; HRSP12-RNase/MRP) and binding of mRNAs could provide further insight into the role of YTHDF2 [49,50].

The broader implications of m^6^A modifications suggest a critical role for epigenetic regulation in mediating adaptive responses to environmental stress. Although specific metabolic data for *X. laevis* under dehydration stress remain limited, studies in other species illustrate how organisms dynamically adjust their metabolism in response to environmental challenges. For example, invasive and native freshwater turtles exhibit distinct metabolic responses to thermal stress that could mirror metabolic shifts in *X. laevis* under dehydration [51]. Similarly, arid-adapted lizards engage in metabolic adjustments that mitigate oxidative stress, indicating that metabolic regulation is likely a conserved mechanism facilitating resilience to diverse stressors [52].

The roles of m^6^A in stabilizing and modulating transcript translation under stress are integral to these responses, yet recent findings highlight that m^6^A may operate alongside other epigenetic mechanisms. Beyond m^6^A, other RNA modifications, such as m1A and m5C, contribute to gene expression regulation under stress, likely acting in concert with m^6^A to fine-tune transcript stability and translation in a reversible, stress-responsive manner [53]. This interplay of epigenetic marks may provide organisms with a flexible regulatory network, enabling rapid physiological shifts critical for survival under environmental pressures. Further studies are warranted to explore the potential synergistic roles of m^6^A and other RNA modifications in mediating adaptive responses, offering a broader perspective on epigenetic contributions to the resilience of *X. laevis* and other amphibians amidst changing environmental conditions [54].

## 5. Conclusions

The findings of this study illustrate a significant downregulation of proteins involved in the m^6^A methylation pathway, which correlates with a strategic reduction in protein synthesis during dehydration in *Xenopus laevis*. Notably, the maintenance of proteins like m^6^A “reader” YTHDF2 highlights the complex regulatory mechanisms that govern mRNA stability and turnover in response to environmental stressors. The substantial increase in total m^6^A levels in the liver and kidneys of dehydrated frogs underscores the critical role of m^6^A methylation as a mechanism facilitating the metabolic rearrangements necessary for survival under extreme dehydration conditions. The observed upregulation of m^6^A in dehydrated tissues suggests that this modification may serve as a rapid, energy-efficient strategy for fine-tuning gene expression, allowing the organism to conserve resources while ensuring the preservation of vital cellular functions. This adaptive response aligns with previous research on other stress-tolerant species, which similarly exhibit stringent regulation of mRNA methylation to optimize energy use and maintain cellular integrity during periods of metabolic rate depression (MRD). To further elucidate the role of m^6^A methylation in stress responses, future studies should investigate its direct interactions with stress-responsive genes in *Xenopus laevis*. Additionally, a comprehensive analysis of other post-transcriptional modifications during dehydration could unveil a more intricate regulatory network, providing deeper insights into the molecular adaptations that enable this amphibian to thrive in fluctuating environments.

## Data Availability

The data that support the findings of this study are available from the corresponding author upon reasonable request.

## Appendix A

**Table A1:** Summary of primary antibody information.

Antibody	Company	Catalogue #
ALKBH5	Abbexa	Abx125516
FTO	Abbexa	Abx125855
METTL14	Abbexa	Abx123810
WTAP	Cell Signalling	56501T
YTHDF1	Abclonal	A13260
YTHDF2	Abclonal	A15616
YTHDF3	Abclonal	A8395
SRSF3	Abclonal	A6067
m^6^A	Abclonal	A19841
